# Novel 5-(nitrothiophene-2-yl)-1,3,4-Thiadiazole Derivatives: Synthesis and Antileishmanial Activity against promastigote stage of Leishmania major

**DOI:** 10.22037/ijpr.2019.14547.12476

**Published:** 2019

**Authors:** Seyed Esmail Sadat-Ebrahimi, Maryam Mirmohammadi, Zahra Mojallal Tabatabaei, Marjan Azimzadeh Arani, Sogol Jafari-Ashtiani, Mahsa Hashemian, Parham Foroumadi, Azadeh Yahya-Meymandi, Setareh Moghimi, Mohammad Hassan Moshafi, Peiman Norouzi, Susan Kabudanian Ardestani, Alireza Foroumadi

**Affiliations:** a *Department of Medicinal Chemistry, Faculty of Pharmacy, Tehran University of Medical Sciences, Tehran, Iran. *; b *Department of Biochemistry, Institute of Biochemistry and Biophysics, University of Tehran, Tehran, Iran. *; c *Drug Design and Development Research Center, The Institute of Pharmaceutical Sciences (TIPS), Tehran University of Medical Sciences, Tehran, Iran. *; d *Pharmaceutics Research Center, Institute of Neuropharmacology, Kerman University of Medical Sciences, Kerman, Iran.*

**Keywords:** Leishmaniasis, Promastigote, 1, 3, 4-thiadiazole, MTT assay, Synthesis

## Abstract

In this study, a series of novel compounds based on 5-(5-nitrothiophene-2-yl)-1,3,4-thiadiazole possessing (het) aryl thio pendant at C-2 position of thiadiazole ring is developed and evaluated as antileishmanial agents using MTT colorimetric assay. 10 New compounds containing aryl and heteroaryl derivatives, started from thiophene-2-carbaldehyde in five steps, were synthesized in good to excellent yields and characterized by ^1^H-NMR, ^13^C-NMR, and IR spectroscopy. Through the compounds **6a-j**, methylimidazole containing derivative **6e** was recognized as the most active compound against *L. major* promastigotes exhibiting IC_50 _values of 11.2µg/mL and 7.1µg/mL after 24 and 48 h, respectively. This compound is > 4 fold more effective than Glucantime as a standard drug (IC_50 _= 50 µg/mL after 24 h and 25 µg/mL after 48 h).

## Introduction

Leishmaniasis, a neglected tropical disease (NTD), is defined as the array of vector-born infections, caused by protozoans of the genus leishmania. The leishmania parasites are transmitted to humans by the bite of female phlebotomine sandflies (1-3).

Leishmaniasis is one of the world’s health problems, causes considerable morbidity, which could be fatal when untreated. According to WHO data, this parasitic disease affects about 350 million people in 88 countries and two million new cases occur yearly (4). Several clinical manifestations are categorized in terms of leishmaniasis. The three main forms are: visceral leishmaniasis, cutaneous leishmaniasis, and muco-cutaneous leishmaniasis (5-7).

Despite great efforts in leishmaniasis treatment, some deficiencies in control programs still need to be made over. No functional vaccine to prevent any form of leishmaniasis has not proved itself and the conventional drugs, including sodium stibogluconate (pentostam) and meglumine antimonate (glucantime) used since 1940 as the linchpins of antileishmanial therapy, exhibit the disadvantages of high toxicity, long half-life, possible therapeutic failure, and unaffordable costs. Therefore, there is a strong demand to find new, safe, and inexpensive potent compounds (8-13).

The nitroheterocycles such as nitrothiophene and nitrofuran have occupied a special place in medicinal chemistry with antiprotozoal and antibacterial properties (14-15). Moreover, lots of 2,5-disubstituted-1,3,4-thiadiazole derivatives have been considerably evaluated in the treatment of leishmaniasis and the results revealed a significant inhibitory activity against leishmaniasis, depending upon the type and position of heterocyclic substituent on thiadiazole ring. It is worth mentioning that among the thiadiazoles with impressive bioactivities, those having a nitroheterocycle nucleus attached to a thiadiazole system have demonstrated unique antiparasitic and antimicrobial activity (16-27).

Considering the pharmacological importance of thiadiazoles and as a part of our continuous efforts pointed out the significance of 2,5-disubstituted-1,3,4-thiadiazole derivatives as anti-parasitic agents, we decided to report the synthesis of new 5-(5-nitrothiophen-2-yl)-1,3,4-thiadiazoles possessing (het) arylthio substituents at C-2 position of thiadiazole ring to assess their antileishmanial activity against the promastigote form of Leishmania major.

## Experimental

*Chemistry*

All the chemicals were obtained from Merck (Germany). Melting point was determined on a Kofler hot stage apparatus (C. Reichert, Vienna, Austria) and uncorrected. The IR spectra were recorded on a shimadzu 470 spectrophotometer, (KBr disks). The ^1^H and ^13^C NMR spectra were measured using Bruker 500 and 125 MHz spectrometer. All NMR samples were run in DMSO solvent and chemical shifts are expressed as parts per million with Me_4_Si as the internal standard. The mass spectra were run on an Agilent 5973 mass spectrometer at 70 eV.

*General procedure for the synthesis of compounds* ***6a-j***

To a solution of 2-chloro-5-(5-nitrothiophene-2-yl) 1,3,4-thiadiazole **5a-j** (1.0 mmol) and K_2_CO_3_ (1.0 mmol) in DMF (7 mL), thiol derivatives (1.2 mmol) were added and the reaction mixture was stirred at 60 °C for 4-5 h. Then, the reaction was allowed to stir at room temperature overnight. The progress of the reaction was monitored by TLC, using petroleum ether/EtOAc as the mobile phase. After completion of the reaction, water (2 mL) was added and the precipitate was filtered off, washed with water and recrystallized with ethanol to give compounds **6a-j**. 

*2-(5-Nitrothiophen-2-yl)-5-(phenylthio)-1,3,4-thiadiazole* (**6a**). Yield: (71%). m.p. 147-149 °C. IR (KBr, cm^–1^): ν = 1512, 1413, 1347, 1326, 1080, 828, 745. ^1^H NMR (500 MHz, DMSO-*d*_6_): δ = 7.57-7.61 (m, 3H), 7.80-7.82 (m, 3H), 8.16 (d, *J* = 4.5 Hz, 1H) ppm. ^13^C NMR (125 MHz, DMSO-*d*_6_): δ = 129.4, 129.7, 130.3, 130.4, 130.5, 134.2, 137.3, 160.5, 167.2, 170.7 ppm.

*2-(p-Tolylthio)-5-(5-nitrothiophen-2-yl)-1,3,4-thiadiazole* (**6b**). Yield: (81%). m.p. 168-169 °C. -IR (KBr, cm^–1^): ν = 1509, 1492, 1467, 1347, 1373, 1081, 814, 729. ^1^H NMR (500 MHz, DMSO-*d*_6_): δ = 2.39 (s, 3H), 7.39 (d, *J* = 8.0 Hz, 2H), 7.69 (d, *J* = 8.0 Hz, 2H), 7.80 (d, *J* = 4.5 Hz, 1H), 8.14 (d, *J* = 4.5 Hz, 1H) ppm. ^13^C NMR (125 MHz, DMSO-*d*_6_): δ = 20.8, 125.8, 130.2, 130.4, 130.5, 131.1, 131.3, 134.4, 137.3, 141.3, 160.2 ppm. MS (m/z, %): 335 (M+, 98), 154 (68), 123 (44), 105 (100), 91 (66), 65 (40), 45 (37).

*2-(4-Chlorophenylthio)-5-(5-nitrothiophen-2-yl)-1,3,4-thiadiazole* (**6c**). Yield: 0.26 g (73%). m.p. 180-182 °C. IR (KBr, cm^–1^): ν = 1508, 1475, 1420, 1371, 1346, 1081, 828. ^1^H NMR (500 MHz, CDCl_3_): δ = 7.28 (d, *J* = 4.0 Hz, 1H), 7.46 (d, *J* = 8.5 Hz, 2H), 7.65 (d, *J* = 8.5 Hz, 2H), 7.86 (d, *J* = 4.0 Hz, 1H) ppm. ^13^C NMR (125 MHz, CDCl_3_): δ = 112.5, 113.1, 122.9, 124.0, 130.7, 135.8, 145.7 (2C), 153.2, 164.9 ppm.

*2-(4-Bromophenylthio)-5-(5-nitrothiophen-2-yl)-1,3,4-thiadiazole* (**6d**). Yield: (65%). m.p. 181-183 °C. IR (KBr, cm^–1^): ν = 1507, 1469, 1420, 1373, 1264, 1011, 824. ^1^H NMR (500 MHz, CDCl_3_): δ = 7.28 (d, *J* = 4.5 Hz, 1H), 7.58 (d, *J* = 8.5 Hz, 2H), 7.62 (d, *J* = 8.5 Hz, 2H), 7.86 (d, *J* = 4.5 Hz, 1H) ppm. ^13^C NMR (125 MHz, CDCl_3_): δ = 112.5, 113.1, 122.5, 124.8, 133.7, 135.9, 145.0, 152.7, 159.0, 162.0.

*2-(1-Methyl-1H-imidazol-2-ylthio)-5-(5-nitrothiophen-2-yl)-1,3,4-thiadiazole* (**6e**). Yield: (70%). m.p. 173-175 °C. IR (KBr, cm^–1^): ν = 1543, 1461, 1411, 1339, 1281, 1225, 730. ^1^H NMR (500 MHz, DMSO-*d*_6_): δ = 3.76 (s, 3H), 7.22 (s, 1H), 7.59 (s, 1H), 7.86 (d, *J* = 5.5 Hz, 1H), 8.17 (d, *J* = 5.5 Hz, 1H) ppm. ^13^C NMR (125 MHz, DMSO-*d*_6_): δ = 126.3, 129.6, 130.4, 130.5, 131.3, 137.2, 152.1, 161.3, 167.8 ppm.

*2-(4,5-Dihydrothiazol-2-ylthio)-5-(5-nitrothiophen-2-yl)-1,3,4-thiadiazole* (**6f**). Yield: (78%). m.p. 249-250 °C. IR (KBr, cm^–1^): ν = 1500, 1449, 1416, 1382, 1344, 1266, 1070, 820. ^1^H NMR (500 MHz, DMSO-*d*_6_): δ = 3.73 (t,* J* = 8.0 Hz, 2H), 5.04 (t, *J* = 8.0 Hz, 2H), 7.91 (d, *J* = 4.0 Hz, 1H), 7.20 (d, *J *= 4.0 Hz, 1H) ppm. ^13^C NMR (125 MHz, DMSO-*d*_6_): δ = 28.2, 58.1, 125.2, 131.5, 134.0, 139.1, 151.4, 157.6, 163.2 ppm.

*2-(5-(5-Nitrothiophen-2-yl)-1,3,4-thiadiazol-2-ylthio)-1H-benzo[d]imidazole* (**6g**). Yield: (70%). m.p. 223-225 °C. IR (KBr, cm^–1^): ν = 1504, 1462, 1361, 1265, 729. ^1^H NMR (500 MHz, DMSO-*d*_6_): δ = 7.28-7.31 (m, 2H), 7.61-7.63 (m, 2H), 7.94 (d, *J* = 4.5 Hz, 1H), 8.20 (d, *J *= 4.5 Hz, 1H), 13.02 (m, NH) ppm. ^13^C NMR (125 MHz, DMSO-*d*_6_): δ = 114.4, 114.5, 115.1, 122.0, 122.7, 122.9, 123.6, 139.1, 141.7, 145.7, 152.2, 157.6, 163.2 ppm.

*2-(5-(5-Nitrothiophen-2-yl)-1,3,4-thiadiazol-2-ylthio)-6-chloro-1H-benzo[d]imidazole* (**6h**). Yield: (70%). m.p. 243-245 °C. IR (KBr, cm^–1^): ν = 1507, 1435, 1361, 1335, 1235, 810, 729. ^1^H NMR (500 MHz, DMSO-*d*_6_): δ = 7.30 (dd, *J *= 8.5, 1.5 Hz, 1H), 7.63 (d, *J *= 8.5 Hz, 1H), 7.70 (d, *J* = 1.5 Hz, 1H), 7.94 (d, *J* = 4.5 Hz, 1H), 8.20 (d, *J* = 4.5 Hz, 1H) ppm. ^13^C NMR (125 MHz, DMSO-*d*_6_): δ = 114.4, 114.5, 115.2, 123.1, 123.3, 127.4, 137.6, 143.6, 145.6 (2C), 152.3, 158.0, 162.5 ppm. MS (m/z, %): 397 (M+2, 45), 395(M+, 100), 319 (69), 241 (12), 183 (49), 156 (35), 124 (21).

*2-(5-(5-Nitrothiophen-2-yl)-1,3,4-thiadiazol-2-ylthio)benzo[d]oxazole *(**6i**). Yield: (75%). m.p. 196-198 °C. IR (KBr, cm^–1^): ν =1500, 1449, 1416, 1344, 1316, 1266, 1071, 820. ^1^H NMR (500 MHz, DMSO-*d*_6_): δ = 7.44-7.49 (m, 2H), 7.79 (d, *J* = 7.0 Hz, 2H), 8.04 (d, *J* = 4.5 Hz, 1H), 8.24 (d,* J* = 4.5 Hz, 1H) ppm. ^13^C NMR (125 MHz, DMSO-*d*_6_): δ = 125.5, 125.9, 126.2, 130.4, 130.5, 130.9, 131.1, 136.7, 140.6, 151.6, 157.3, 159.0, 163.9 ppm.

*2-(5-(5-Nitrothiophen-2-yl)-1,3,4-thiadiazol-2-ylthio)benzo[d]thiazole* (**6j**). Yield: (68%). m.p. 215-217 °C. IR (KBr, cm^–1^): ν = 1509, 1411, 1338, 1258, 1066, 761, 729. ^1^H NMR (500 MHz, DMSO-*d*_6_): δ = 7.52 (t, *J* = 7.5 Hz, 1H), 7.60 (t, *J* = 7.5 Hz, 1H), 8.01 (d, *J *= 3.5 Hz, 1H), 8.07 (d, *J* = 8.0 Hz, 1H), 8.16 (d, *J* = 8.0 Hz, 1H), 8.24 (d, *J *= 3.5 Hz, 1H) ppm.

*In-vitro antileishmanial activity*

Antileishmanial effects of compounds on promastigote were investigated by 3-(4,5-dimethylthiazol-2-yl)-2,5-diphenyltetrazolium bromide (MTT) colorimetric assay. All of the compounds were screened at two concentrations 10 and 20 μg/mL. The 50% inhibitory concentration (IC_50_) was calculated using various concentrations (1, 5, 10 and 20 μg/mL) of most active compound **6e**, Log phase parasites (6 ×10^7^ cells/mL) were incubated with compounds for 24 h at the room temperature. The positive and negative controls were Glucantime and DMSO as respectively. After 24 h, the media was removed and the cells were treated with MTT solution (5 mg/mL) for 4 h at 37 ^°^C. Then, the cells were centrifuged for 15min at 2000 rpm and the pellets were dissolved in 100µL of DMSO and the absorbances were read in a microplate reader set at a wavelength of 492 nm.

**Figure1:**
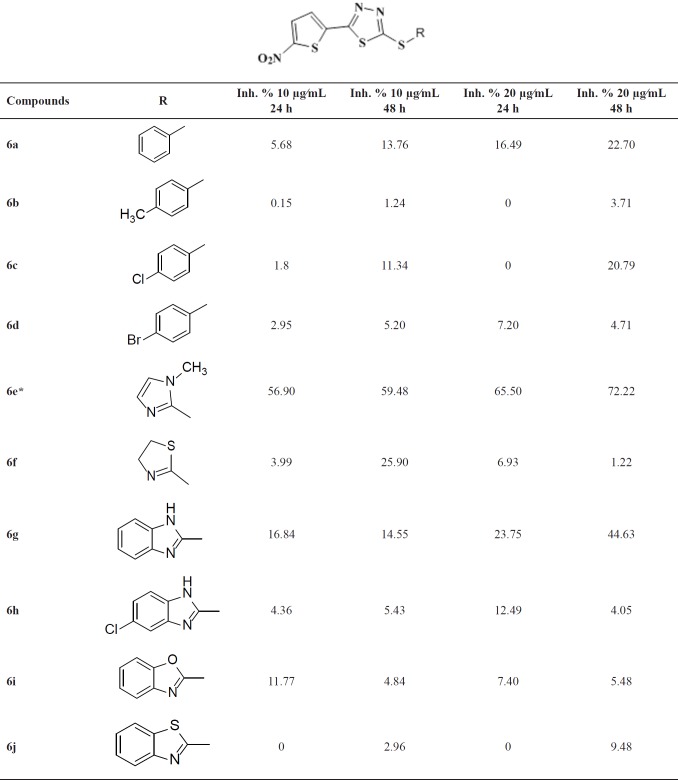
Synthesis of compounds **6a-j**. Reagents and conditions: (i) HNO_3_, AC_2_O, CH_3_COOH; (ii) thiosemicarbazide, EtOH, HCl, reflux; (iii) NH_4_Fe (SO_4_)_2_.12H_2_O, reflux; (iv) NaNO_2_, HCl, Cu; (v) Ar-SH, K_2_CO_3_, DMF, 60 °C

**Table1:** Structure and *in-vitro* inhibitory activities of compounds **6a-j** against the promastigote form of *L. major*

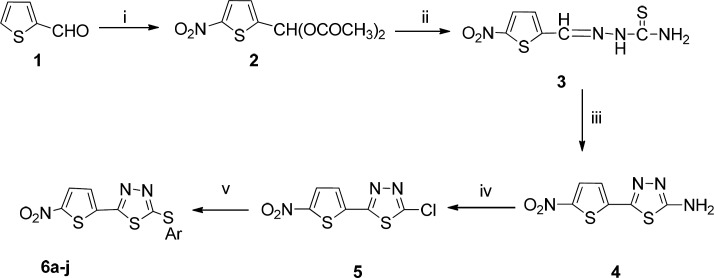

* IC_50_ for **6e**: 11.2 µg/mL after 24 h and 7.1 µg/mL after 48 h,

IC_50 _for Glucantim: 50 µg/mL after 24 h and 25 µg/mL after 48 h.

## Results and Discussion

*Chemistry*

The synthetic strategy to achieve target compounds **6a-j** is outlined in Figure 1. The key intermediate compound **5 **was obtained from thiophene-2-carboxaldehyde in four steps according to our previously reported synthetic method (28). 

The reaction of 2-chloro-5-(5-nitrothiophene-2-yl)1,3,4-thiadiazole **5** with appropriate thiol derivatives in the presence of potassium carbonate in dimethyl formamide (DMF) at 60 °C afforded title compounds **6a-j** in good yields.

*Pharmacology*

In our previous work, we studied some 5-(nitroaryl)-1,3,4- thiadiazoles bearing different side chains such as phenacylthio, benzoylethanethio, and benzylthio derivatives on C-2 position of thiadiazole (29), obtaining promising results. So, we intended to develop new 2-(het) arylthio-5-(nitrothiophen)-1,3,4-thiadiazole analogous in order to investigate the antileishmaniasis activity of the compounds with S-pendant groups. For this purpose, we synthesized and evaluated anti-parasitic activity of the title compounds **6a-j** against leishmania promastigote using MTT assay. 

The *in-vitro* anti-leishmania activities of the compounds were investigated at 10 and 20 µg/mL. The results are listed in Table 1. Compared to our previous findings, the observed weak inhibitory activities of the target compounds revealed the importance of alkyl linker in S-pendant groups.

Among the synthesized compounds, Benzimidazole containing derivative **6g** was more active than the corresponding benzoxazole **6i** and benzothiazole **6j** analogues. Unsubstituted aryl derivatives exhibited better activity compared to the substituted ones. (Compound **6a** versus **6b**, **6c** and** 6d** and compound **6g** versus **6h**). 

Methyl-imidazole containing derivative **6e** with IC_50 _= 11.2 µg/mL, after 24 h and 7.1 µg/mL after 48 h, shows the most potent activity in preventing the growth of promastigotes. This compound exhibits better performance in comparison to glucantime as a reference drug. The IC_50 _of glucantime was 50 µg/mL after 24 h and 25 µg/mL after 48 h. The inhibitory concentrations for 50% of inhibition (IC_50_) of parasitic growth after 24 and 48 h of the incubation were calculated based on a linear regression and reported as a mean.

## Conclusion

In conclusion, a series of novel 5-(5-nitrothiophen-2-yl)-1,3,4-thiadiazoles possessing (het) arylthio substituent at C-2 position of thiadiazole ring are synthesized and screened for inhibitory activity against promastigote form of *L. major*. Methyl-imidazolyl containing compound, **6e**, was identified as the most potent compound against leishmaniasis which significantly reduced the viability of *L. major* promastigote.
